# A Two-Stage Approach for Bayesian Joint Models of Longitudinal and Survival Data: Correcting Bias with Informative Prior

**DOI:** 10.3390/e23010050

**Published:** 2020-12-31

**Authors:** Valeria Leiva-Yamaguchi, Danilo Alvares

**Affiliations:** Department of Statistics, Pontificia Universidad Católica de Chile, Macul, Santiago 7820436, Chile; vjleiva@mat.uc.cl

**Keywords:** Bayesian inference, bias reduction, individual fixed-effect, Stan

## Abstract

Joint models of longitudinal and survival outcomes have gained much popularity in recent years, both in applications and in methodological development. This type of modelling is usually characterised by two submodels, one longitudinal (e.g., mixed-effects model) and one survival (e.g., Cox model), which are connected by some common term. Naturally, sharing information makes the inferential process highly time-consuming. In particular, the Bayesian framework requires even more time for Markov chains to reach stationarity. Hence, in order to reduce the modelling complexity while maintaining the accuracy of the estimates, we propose a two-stage strategy that first fits the longitudinal submodel and then plug the shared information into the survival submodel. Unlike a standard two-stage approach, we apply a correction by incorporating an individual and multiplicative fixed-effect with informative prior into the survival submodel. Based on simulation studies and sensitivity analyses, we empirically compare our proposal with joint specification and standard two-stage approaches. The results show that our methodology is very promising, since it reduces the estimation bias compared to the other two-stage method and requires less processing time than the joint specification approach.

## 1. Introduction

Joint models of longitudinal and survival data have been an essential statistical tool in medical research [1,2]. This class of models became popular due to its ability to provide complete inference (longitudinal, survival, and association between both of them), reduce estimation bias, increase statistical efficiency, and conveniently make predictions of outcomes [3,4,5]. However, there ain’t no such thing as a free lunch. The complexity of these models makes the computational process quite demanding and sometimes impractical.

In this paper, we focus on general contexts in which longitudinal measurements are observed strictly before the survival time [6]. This framework has been analysed in several applications, see References [7,8,9] for a review on joint models up to date, and it has at least two drawbacks: (i) identifiability problems due to the large number of parameters [7,10,11,12,13] and (ii) requirement for numerical integrations that can make the inferential process time-consuming [14,15,16,17,18].

Two-stage approaches alleviate both problems that arise with simultaneous inference for joint models [19,20]. Typically, the two-stage approach fits the longitudinal submodel first and then uses the estimated parameters to approximate the longitudinal trajectory, as an endogenous time-varying covariate, within the survival submodel. This strategy is usually simple to implement and allows us to use flexible models available in standard longitudinal and survival analyses packages (separately). In the current literature of joint models, there are different proposals for two-stage methods in both frequentist [20,21,22,23,24] and Bayesian [25,26,27] approaches. These two-stage procedures speed up processing time by estimating two less complex submodels than the joint model. However, the main weakness of this methodology is that by ignoring the joint nature between both processes, the estimates of the survival regression parameters are often biased [22,28,29,30].

From a Bayesian perspective, we work around this problem by proposing a two-stage approach that, after fitting the longitudinal submodel, corrects bias through an individual and multiplicative fixed-effect with highly informative prior inserted in the survival submodel.

The paper is organised as follows—Section 2 introduces a general formulation of joint models. Section 3 and Section 4 describe the standard joint and two-stage approaches. Section 5 presents our two-stage strategy. Section 6 validates and compares the performance of our proposal against the other standard approaches. Finally, Section 7 discusses the advantages, limitations and extensions of our methodology. Appendix A and Appendix B show sensitivity analyses and other simulated scenarios.

## 2. Bayesian Joint Model Formulation

We assume that there are *n* individuals with repeated measures and time to an event of interest individually associated. In particular, underlying characteristics from the longitudinal process, which models repeated measures, are shared with the time-to-event process [30].

### 2.1. Longitudinal Submodel

We use the well-known linear mixed-effects specification to model the repeated measures over time [31,32]. In this case, the response variable $y_{i}\left(t\right)$ of individual *i* at time *t* is given by:

(1) $$\begin{array}{cc} y_{i}\left(t\right) & =\mu_{i}\left(t\right)+\epsilon_{i}\left(t\right)=x_{L,i}^{⊤}\left(t\right)\beta +z_{i}^{⊤}\left(t\right)b_{i}+\epsilon_{i}\left(t\right),\\ b_{i} & \overset{i.i.d.}{∼}N\left(0,\Sigma \right)\;\mathrm{and}\;\epsilon_{i}\left(t\right)\overset{i.i.d.}{∼}N\left(0,\sigma^{2}\right), \end{array}$$

where the true unobserved value of the longitudinal outcome at time *t*, $\mu_{i}\left(t\right)$, is characterised by the linear combination between the covariate vectors, $x_{L,i}\left(t\right)$ and $z_{i}\left(t\right)$, and their respective fixed (β) and random ($b_{i}$) effect vectors; $b_{i}$ represents the vector of individual random effects with a K×K variance-covariance matrix Σ, where *K* is the number of random effects; and $\epsilon_{i}\left(t\right)$ denotes the measurement error term with variance $\sigma^{2}$.

### 2.2. Survival Submodel

The proportional hazards specification is widely used to model this type of problem [33]. Let $T_{i}^{*}$ denote the event time for individual *i*, $C_{i}$ the censoring time, $T_{i}=\min\left\{T_{i}^{*},C_{i}\right\}$ the observed time, and $\delta_{i}=I\left(T_{i}^{*}\leq C_{i}\right)$ the event indicator. So, the hazard function of the survival time $T_{i}$ of individual *i* is expressed by:

(2) $$h_{i}\left(t|M_{i}\left(t\right)\right)=h_{0}\left(t\right)\exp\left\{x_{S,i}^{⊤}\gamma +\alpha \mu_{i}\left(t\right)\right\},$$

where $h_{0}\left(t\right)$ represents an arbitrary baseline hazard function at time *t* and $x_{S,i}$ is a covariate vector with coefficients γ. $M_{i}\left(t\right)=\left\{\mu_{i}\left(l\right),\;0\leq l<t\right\}$ denotes the history of the longitudinal process up to *t*; $\mu_{i}\left(t\right)$ is defined as in (1) and has the role of connecting both processes, while α measures the strength of this association. In order to simplify the notation, we will omit the term $M_{i}\left(t\right)$ when specifying a hazard function.

### 2.3. Prior Distributions

To complete the Bayesian joint model formulation, we have to assign prior distributions to all parameters and hyperparameters. As a standard specification, we assume independent and diffuse prior, that is, proper distributions with a large variance [34]. More specifically, β, γ and α follow Normal distributions with mean at zero and large variance; σ follows a weakly-informative half-Cauchy(0,5) [35]; and Σ follows an inverse-Wishart(V,r), where *V* is a K×K identity matrix, r=K is the degrees-of-freedom parameter [36]. Once the baseline hazard function $h_{0}\left(t\right)$ is defined, diffuse priors are also specified for its parameters.

## 3. Joint Specification (JS) Approach

Let y and s be the longitudinal and survival data, respectively. The vector of all parameters and hyperparameters is specified by θ and the random effects by b. So, the full joint distribution of (y,s,b,θ) can be factorised as the product of the joint conditional distribution f(y,s∣b,θ), the conditional distribution of the random effects f(b∣θ), and the prior distribution π(θ). Equationally,

(3) f(y,s,b,θ)=f(y,s∣b,θ)f(b∣θ)π(θ).

There are different proposals for the specification of the conditional distribution f(y,s∣b,θ) [37]. However, the most widely used approach is the shared-parameter specification [38], in which it assumes that the longitudinal process is conditionally independent of the survival process given the shared information:

(4) f(y,s∣b,θ)=f(y∣b,θ)f(s∣b,θ),

where f(y∣b,θ) and f(s∣b,θ) are commonly specified according to the joint models (1) and (2).

From a joint approach, the inferential procedure to estimate (b,θ) based on Equations (3) and (4) should be performed simultaneously. In addition, this joint modelling is usually quite complex due to the high number of parameters and potential integrations with no closed-form derived from the calculation of the survival function obtained from Equation (2). Hence, as expected, the processing of the inferential procedure is very time-consuming.

## 4. Standard Two-Stage (STS) Approach

Two-stage strategies are very useful for reducing the complexity of joint models and speeding up the inferential process. From a frequentist point of view, Tsiatis et al. [20] proposed one of the most popular two-stage approaches. The first stage is to fit the longitudinal submodel (1) and then the trajectory function $\mu_{i}\left(t\right)$ is calculated using the estimated parameters and random effects. In the second stage, this trajectory function estimated is considered as an endogenous time-varying covariate when fitting the survival submodel (2).

As a potential competitor, we use the Tsiatis et al. [20] approach adapted to the Bayesian framework. Specifically, in the first stage, we calculate the posterior mean of the longitudinal submodel parameters and random effects shared with the survival submodel, that is, $\hat{\beta }=E\left(\beta |y\right)$ and $\hat{b}=E\left(b|y\right)$. In the second stage, we incorporate the trajectory function into the survival submodel considering ${\hat{\mu }}_{i}\left(t\right)=x_{L,i}^{⊤}\left(t\right)\hat{\beta }+z_{i}^{⊤}\left(t\right){\hat{b}}_{i}$, for i=1,…,n, and then the posterior distribution of $\left(\gamma ,\alpha ,h_{0}\right)$ is calculated.

## 5. Novel Two-Stage (NTS) Approach

The first part of our two-stage proposal is similar to the STS approach, that is, the posterior distributions of the longitudinal submodel parameters and random effects are calculated. However, we propose the following modification to the survival submodel:

(5) $$h_{i}\left(t\right)=w_{i}\;h_{0}\left(t\right)\exp\left\{x_{S,i}^{⊤}\gamma +\alpha {\hat{\mu }}_{i}\left(t\right)\right\},$$

where $w_{i}>0$ denotes a multiplicative fixed-effect for individual *i* and ${\hat{\mu }}_{i}\left(t\right)$ is calculated in the same way as the standard two-stage approach.

The role of $w_{i}$ is essential to satisfactorily correct the estimation bias by ignoring the potential joint nature between both processes. In addition, this term can also correct problems of model misspecification and unobserved heterogeneity [39]. Specifically, what we propose is a very small perturbation using an individual fixed-effect. Hence, to do that, we specify a highly informative prior distribution for $w_{i}$, given by:

(6) $$w_{i}∼\mathrm{Gamma}\left(\eta ,\eta \right),$$

where $E(w_{i})=1$ and η is a known parameter and must be specified such that $\mathrm{Var}(w_{i})=1/\eta$ is small. Interpretatively, if $w_{i}$ is not perturbed (i.e., $\mathrm{Var}(w_{i})=0$), then we turn to the standard two-stage approach presented in Section 4. Moreover, note that if we assume that η is an unknown parameter and so a hyperprior should be set for it, then the specification (5) becomes a Bayesian frailty model [40]. In practice, the latter option is convergently unstable and therefore will not be addressed in this paper.

In the context of frailty models, $w_{i}$ is typically modelled through a Gamma distribution [41]. For this reason, we chose such distribution in (6). However, other non-negative continuous distributions could be used as long as $E(w_{i})=1$ is satisfied.

## 6. Simulation Study

To evaluate whether the novel two-stage approach reduces the bias with low computational time, we perform a simulation study that compares our proposal with the joint specification (see Section 3) and standard two-stage (see Section 4) approaches.

The joint formulation that is considered here is based on submodels (1) and (2). In particular, the longitudinal specification for individual *i* at time *t* is given by:

(7) $$\begin{array}{cc} y_{i}\left(t\right) & =\mu_{i}\left(t\right)+\epsilon_{i}\left(t\right)=\beta_{0}+b_{0i}+\left(\beta_{1}+b_{1i}\right)t+\beta_{2}x_{i}+\epsilon_{i}\left(t\right),\\ b_{i} & ={\left(b_{0i},b_{1i}\right)}^{⊤}\overset{i.i.d.}{∼}N\left(0,\Sigma \right)\;\mathrm{and}\;\epsilon_{i}\left(t\right)\overset{i.i.d.}{∼}N\left(0,\sigma^{2}\right), \end{array}$$

where the covariate $x_{i}$ is a binary group indicator simulated from a Bernoulli distribution with probability 0.5 and will be called group parameter.

Based on the simulation scenarios proposed by Furgal et al. [8], we adopt the following hazard specification for individual *i*:

(8) $$h_{i}\left(t\right)=\exp\left\{\gamma_{0}+\gamma_{1}x_{i}+\alpha \mu_{i}\left(t\right)\right\},$$

where the baseline hazard function has an exponential specification, $h_{0}\left(t\right)=\exp\left(\gamma_{0}\right)$. Note that other options for this function could be preferred, such as Gamma, Weibull, Gompertz, log-normal, log-logistic, piecewise, splines, and so forth [42,43].

### 6.1. Simulating Data for Joint Models

As a preliminary simulation step, all parameters and hyperparameters θ=(β,Σ,σ,γ,α), number of individuals (*n*), minimum number of longitudinal observations ($m_{\min}$), and maximum observational time ($t_{\max}$) must be set. Then, the covariate $x_{i}$ and the random effects $b_{i}$, for i=1,…,n, are simulated.

The true event time for individual *i* is simulated using the well-known inverse transform sampling [44], where $T_{i}^{*}=S_{i}\left(u\right)$, *u* is generated from a standard uniform distribution, and $S_{i}$ denotes the survival function derived from Equation (8). The censoring time for each individual, $C_{i}$, is generated from a uniform distribution on the interval $\left(0,t_{\max}\right)$ and then the observed time is set as $T_{i}=\min\left\{T_{i}^{*},C_{i}\right\}$ and the event indicator as $\delta_{i}=I\left(T_{i}^{*}\leq C_{i}\right)$.

The number of longitudinal observations of individual *i*, $n_{i}$, is set as $m_{\min}$ plus the largest integer less than $T_{i}$ (i.e., $\left\lfloor T_{i}\right\rfloor$). The recording times of the repeated measurements are equispaced set from 0 to $\left\lfloor T_{i}\right\rfloor$. The random errors $\epsilon_{i}\left(t_{1}\right),\ldots ,\epsilon_{i}\left(t_{n_{i}}\right)$ are simulated from a normal distribution with mean at zero and variance $\sigma^{2}$. Finally, the longitudinal observations of individual *i*, $y_{i}\left(t_{1}\right),\ldots ,y_{i}\left(t_{n_{i}}\right)$, are computed according to the submodel (7).

The simulation scheme to jointly generate longitudinal and survival data is summarised in Algorithm 1.
**Algorithm 1:** Simulation scheme**0** Initialisation: Set θ, *n*, $m_{\min}$, and $t_{\max}$.**1** Survival submodel:Simulate $x_{i}∼\mathrm{Bern}\left(0.5\right)$ and $b_{i}∼N\left(0,\Sigma \right)\;\forall i$.  Simulate $T_{i}^{*}$ based on the survival submodel (8) and sample $C_{i}∼U\left(0,t_{\max}\right)\;\forall i$.  Set $T_{i}=\min\left\{T_{i}^{*},C_{i}\right\}$ and $\delta_{i}=I\left(T_{i}^{*}\leq C_{i}\right)\;\forall i$.**2** Longitudinal submodel:Set $n_{i}=m_{\min}+\left\lfloor T_{i}\right\rfloor \;\forall i$.  Set $0=t_{1},\ldots ,t_{n_{i}}=\left\lfloor T_{i}\right\rfloor \;\forall i$ equispaced.  Simulate $\epsilon_{i}\left(t\right)∼N\left(0,\sigma^{2}\right),\;\;t=t_{1},\ldots ,t_{n_{i}}\;\forall i$.  Compute $y_{i}\left(t_{1}\right),\ldots ,y_{i}\left(t_{n_{i}}\right)\;\forall i$ based on the longitudinal submodel (7).

### 6.2. Scenarios

We present simulation scenarios generated from the prothro dataset, which is available in the R-package JM (version 1.4-8) from the CRAN at http://cran.r-project.org/package=JM. This dataset includes 488 patients with histologically verified liver cirrhosis, where 251 patients were randomised to a treatment with prednisone and the remaining received placebo [45]. The longitudinal variable pro is used on a logarithmic scale and the treatment variable (treat) is defined as $x_{i}$ in both submodels.

First, we fit the joint models (7) and (8) for prothro data using the function *jointModel* from the R-package JM. Then, the estimates are used as “true parameter values” in the generation of simulated data, also using the joint formulations (7) and (8). The jointly estimated parameters are ${\hat{\beta }}_{0}$ = 4.274, ${\hat{\beta }}_{1}$ = −0.004, ${\hat{\beta }}_{2}$ = −0.097, $\hat{\sigma }$ = 0.262, ${\hat{\Sigma }}_{11}$ = 0.094, ${\hat{\Sigma }}_{22}$ = 0.005, and ${\hat{\Sigma }}_{12}$ = ${\hat{\Sigma }}_{21}$ = −0.001 for the longitudinal submodel (7); and ${\hat{\gamma }}_{0}$ = 8.671, ${\hat{\gamma }}_{1}$ = −0.172, and $\hat{\alpha }$ = −2.447 for the survival submodel (8). Finally, we simulate 100 datasets with *n*=200,500,1000, $m_{\min}$ = 3, and $t_{\max}$ = 15. Other simulation scenarios are presented in Appendix B.

The Bayesian joint model specification (7) and (8) with the prior distributions presented in Section 2.3 is used for the three estimation strategies. The MCMC configuration is defined as follows: 2000 iterations with warm-up of 1000 for the joint model using the JS approach and for the longitudinal submodel from both two-stage approaches. Additionally, 1000 iterations with warm-up of 500 are set to run the survival submodel from both two-stage approaches. All models were implemented using rstan (http://mc-stan.org) and the codes are provided in a Supplementary Material. Simulations were performed on a Dell laptop with 2.6 GHz Intel Core i7, 16 GB RAM, OS Windows.

Here, the η parameter is set to 1.5 and so the prior variance of $w_{i}$ is equal to 1/1.5≈0.67. Of course, this variance value is small and informative for the scale of simulated data in this paper, but it can still be very large for other problems. A sensitivity analysis for the choice of η is presented in Appendix A.

**Figure 1:**
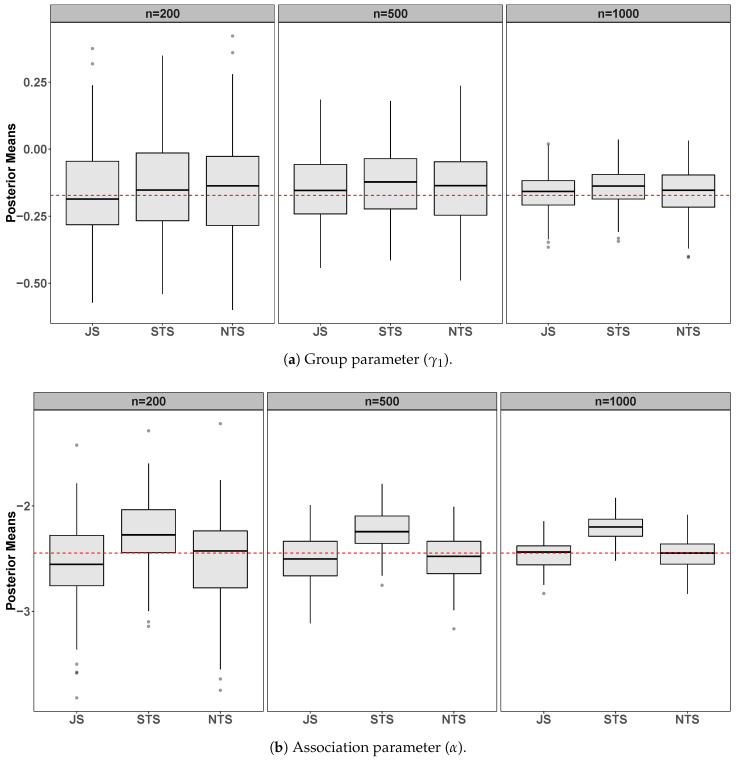
Simulation results from 100 datasets comparing the joint specification (JS), the standard two-stage (STS), and the novel two-stage (NTS) for *n* = 200,500,1000. The panels show posterior means from the 100 datasets for the survival submodel group (**a**) and association (**b**) parameters. The dashed horizontal line indicates the true parameter value.

Table 1 and Figure 1 show the comparative results among joint specification (JS), standard two-stage (STS), and novel two-stage (NTS) approaches for 100 simulated datasets from the joint models (7) and (8) using the parameters set above.

We can see, both in Table 1 and in Figure 1, that the group parameter ($\gamma_{1}$) is satisfactorily estimated using the three approaches. On the other hand, in all scenarios, our approach also estimates the association parameter (α) very well. These results are better than the STS approach and similar to the JS, which in theory is the correct way to deal with the estimation process. However, as expected, the standard deviation of posterior distributions using our methodology is slightly higher than others. Furthermore, the computational time of the NTS is a little higher than of the STS and much less than that of the JS approach.

It is worth noting that theoretically the joint specification approach is always preferable. The other two approaches are recommended when the complexity of the joint model makes the inferential procedure highly time-consuming or when there are problems of convergence of the Markov chains due to the high-dimensional parameter space. In the model selection framework (e.g., variable selection problems or model selection from different hazard function proposals), Bayesian selection criteria can be applied in the usual way. In particular, leave-one-out cross-validation (LOO) and the widely applicable information criterion (WAIC) can be easily calculated using the R-package loo [46] as well as Bayes factors and posterior model probabilities using the R-package bridgesampling [47].

## 7. Discussion

In this paper, we presented a novel two-stage (NTS) method for fitting Bayesian joint models of longitudinal and survival data using fixed-effects with informative prior to correct the estimation bias caused by ignoring the joint nature of both processes. We demonstrated in different scenarios that our proposal is more accurate than the standard two-stage (STS) approach and its processing time is much less than the joint specification (JS) approach.

In our simulation studies, we found that the group parameter estimation from the survival submodel is robustly estimated regardless of the estimation approach. This result was expected since this parameter does not depend on shared information. On the other hand, the association parameter is sensitive when using the STS strategy.

The specification of the informative prior variance for the fixed-effects can be critical drawback of our approach. In our simulation study, the set value produced quite satisfactory results (see the sensitivity analysis of this parameter in Appendix A). However, we would like to reinforce to the reader that this choice depends on the scale of the problem, in which the value used in this paper may not be appropriate in other applications.

It would be interesting to apply the NTS in more complex longitudinal (e.g., skewed or multiple longitudinal data) and survival (e.g., competing-risks or multistate data) submodels than those employed here. Hence, we would be able to try determining the limits of the methodology. Furthermore, our proposal could also be combined with sequential methods for Bayesian joint models [48].

## Figures and Tables

**Table 1:** Posterior summary and computational time (in minutes) from each estimation approach.

Posterior	Parameter	JS			STS			NTS		
	(True Value)	*n* = 200	*n* = 500	*n* = 1000	*n* = 200	*n* = 500	*n* = 1000	*n* = 200	*n* = 500	*n* = 1000
Mean	$\gamma_{1}$ (−0.172)	−0.172	−0.152	−0.165	−0.148	−0.132	−0.144	−0.141	−0.148	−0.160
	α (−2.447)	−2.574	−2.505	−2.460	−2.271	−2.237	−2.204	−2.537	−2.491	−2.456
SD	$\gamma_{1}$	0.193	0.120	0.084	0.184	0.115	0.082	0.241	0.149	0.106
	α	0.345	0.214	0.148	0.295	0.186	0.131	0.391	0.245	0.171
**Average Comp. Time**		4.291	11.055	23.428	1.885	4.692	11.906	2.224	5.836	13.845

**Figure A1:**
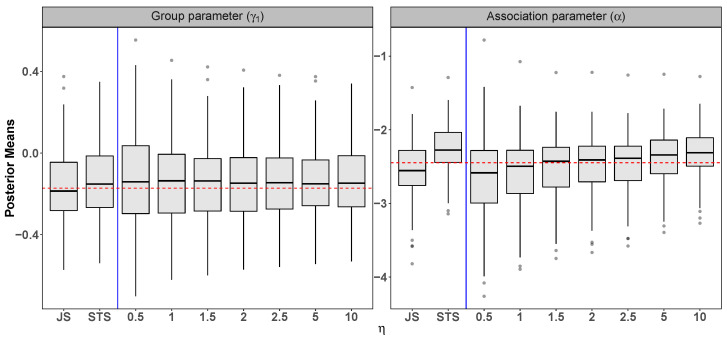
Sensitivity analysis for η from 100 datasets with n=200. Since $\mathrm{Var}(w_{i})=1/\eta$ (for the Gamma prior), larger values of η lead to small perturbation of $w_{i}$’s and therefore the results resemble the standard two-stage (STS) approach. The dashed horizontal line indicates the true parameter value.

**Figure A2:**
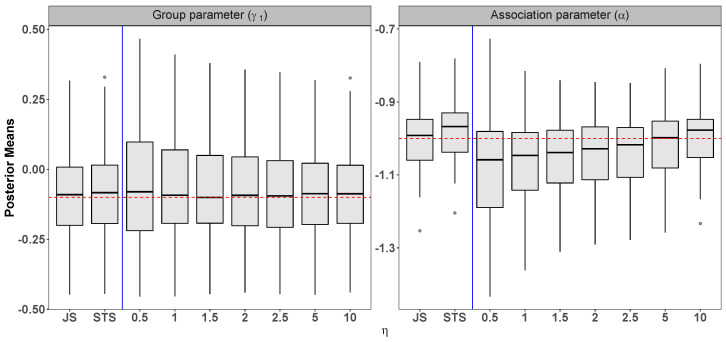
Scenario 1: Simulation results from 100 datasets comparing the joint specification (JS), the standard two-stage (STS), and the novel two-stage (NTS) for η=0.5,1,1.5,2,2.5,5,10. The dashed horizontal line indicates the true parameter value.

**Figure A3:**
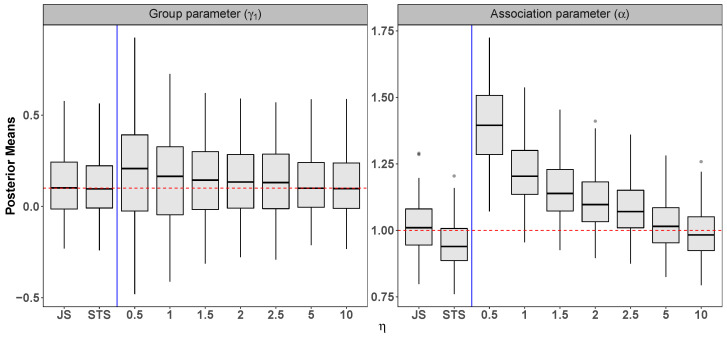
Scenario 2: Simulation results from 100 datasets comparing the joint specification (JS), the standard two-stage (STS), and the novel two-stage (NTS) for η=0.5,1,1.5,2,2.5,5,10. The dashed horizontal line indicates the true parameter value.

## Data Availability

Data sharing not applicable.

## Supplementary Materials

The codes are available online at https://www.mdpi.com/1099-4300/23/1/50/s1.

## Appendix B. Other Simulation Studies

### Appendix B.1. Scenario 1

**Setting:**$\beta_{0}$ = 5, $\beta_{1}$ = −0.02, $\beta_{2}$ = −0.1, σ = 0.25, $\Sigma_{11}$ = 1, $\Sigma_{22}$ = 1, $\Sigma_{12}$ = $\Sigma_{21}$ = 0.2, $\gamma_{0}$ = 5, $\gamma_{1}$ = −0.1, α = −1, *n* = 200, $m_{\min}$ = 3, and $t_{\max}$ = 15.

### Appendix B.2. Scenario 2

**Setting:**$\beta_{0}$ = 1, $\beta_{1}$ = −0.1, $\beta_{2}$ = −0.01, σ = 0.5, $\Sigma_{11}$ = 1, $\Sigma_{22}$ = 1, $\Sigma_{12}$ = $\Sigma_{21}$ = 0.2, $\gamma_{0}$ = −3, $\gamma_{1}$ = 0.1, α = 1, *n* = 200, $m_{\min}$ = 3, and $t_{\max}$ = 15.
